# A Comparative Analysis of Individual RAS Mutations in Cancer Biology

**DOI:** 10.3389/fonc.2019.01088

**Published:** 2019-10-18

**Authors:** Carmen Muñoz-Maldonado, Yitzhak Zimmer, Michaela Medová

**Affiliations:** ^1^Department of Radiation Oncology, Inselspital, Bern University Hospital, Bern, Switzerland; ^2^Radiation Oncology, Department for BioMedical Research, University of Bern, Bern, Switzerland

**Keywords:** RAS mutations, RAS profile, RAS-mutated cancers, treatment responses, RAS-related omics, GTP/GDP binding, RAS signaling, rare codons

## Abstract

In human cells, three closely related *RAS* genes, termed *HRAS, KRAS*, and *NRAS*, encode four highly homologous proteins. RAS proteins are small GTPases involved in a broad spectrum of key molecular and cellular activities, including proliferation and survival among others. Gain-of-function missense mutations, mostly located at codons 12, 13, and 61, constitutively activate RAS proteins and can be detected in various types of human cancers. *KRAS* is the most frequently mutated, followed by *NRAS* and *HRAS*. However, each isoform exhibits distinctive mutation frequency at each codon, supporting the hypothesis that different RAS mutants may lead to distinct biologic manifestations. This review is focused on the differences in signaling and phenotype, as well as on transcriptomics, proteomics, and metabolomics profiles related to individual RAS-mutated variants. Additionally, association of these mutants with particular targeted outcomes and rare mutations at additional RAS codons are discussed.

## Introduction

RAS subfamily comprises the ubiquitously expressed human RAS proteins KRAS4A, KRAS4B (the two KRAS splice variants), HRAS, and NRAS, which are frequently mutated in cancer (1). These genes encode small GTPases that function as molecular regulators, controlling a broad spectrum of cellular activities, such as proliferation and cell survival (2).

RAS proteins are considered molecular switches because they cycle between the “on” and “off” conformations, which are given by the binding of GTP and GDP, respectively (3). The transition between both states is regulated by two different protein families. The guanine nucleotide exchange factors (GEFs) promote GDP dissociation and GTP binding while the GTPase-activating proteins (GAPs) stimulate RAS intrinsic GTPase activity to switch off this signal.

High homology is shared by the three RAS proteins, except for the C-terminus hypervariable region, which is thought to confer the specific function of each protein (2). It has been reported that up to one-third of human cancers (4) bears gain-of-function missense mutations (5) that occur in the protein region that is identical among the four RAS proteins. Forty-four different point mutations have been described and 99.2% of them are located at codons 12, 13, and 61 (2), but other non-canonical codons (such as 19, 117, or 146) are also mutated at low frequencies (6). All these canonical mutations prompt the loss of the intrinsic and/or the GAP-stimulated GTPase activity of RAS proteins, leading to a constitutively activated form of RAS. However, some non-canonical mutations, such as for example HRAS A146 mutations, do not impair RAS GTPase activity, but increase guanine nucleotide exchange.

Interestingly, the mutated isoform, as well as the codon position and the amino acid substitution varies among RAS proteins in human cancers, but the reason remains to be established (4). Regarding protein variability, *KRAS* is the most frequently mutated protein in human cancers, followed by *NRAS* and *HRAS*. Oncogenic alterations in *KRAS* are more frequent in patients with pancreatic carcinoma, colorectal tumors and lung malignancies (5). Mutations in *HRAS* can be found in dermatological malignancies and head and neck cancers, while *NRAS* mutations are common in melanomas and in some hematopoietic malignancies (Table 1) (5).

**Table 1:** Most common mutations in the individual codons of RAS proteins.

**RAS protein**	**Malignancies**	**Codon**	**Amino acid substitution**
HRAS	Dermatological Head and neck cancer	Codon 12: GGC (Gly, G)	12A, 12C, 12D, 12R, 12S, 12V
		Codon 13: GGT (Gly, G)	13C, 13D, 13R, 13S, 13V
		Codon 61: CAG (Gln, Q)	61H, 61K, 61L, 61P, 61R
KRAS	Pancreatic carcinoma Colorectal cancer Lung malignancies	Codon 12: GGT (Gly, G)	12A, 12C, 12D, 12R, 12S, 12V
		Codon 13: GGC (Gly, G)	13A, 13C, 13D, 13R, 13S, 13V
		Codon 61: CAA (Gln, Q)	61E, 61H, 61K, 61L, 61P, 61R
NRAS	Melanomas Hematopoietic malignancies	Codon 12: GGT(Gly, G)	12A, 12C, 12D, 12R, 12S, 12V
		Codon 13: GGT (Gly, G)	13A, 13C, 13D, 13R, 13S, 13V
		Codon 61: CAA (Gln, Q)	61E, 61H, 61K, 61L, 61P, 61R

*Amino acid substitutions identified at codon 12, 13, and 61 of each RAS protein, highlighting in red the most frequently observed. Gly and G, glycine; Gln and Q, glutamine; A, alanine; C, cysteine; D, aspartic acid; R, arginine; S, serine; V, valine; H, histidine; K, lysine; L, Leucine; P, proline; E, glutamic acid*.

The mutations rates at each codon differ between the RAS proteins (2). While *KRAS* is commonly mutated at codon 12 with only few mutations occurring at codon 61, *NRAS* mutations are most frequently observed at codon 61. In addition, *HRAS* mutational rate is similar for both codons 12 and 61, displaying an intermediate mutational pattern between *KRAS* and *NRAS* (2).

Each of these codons can be substituted through a single-nucleotide change resulting in codons 12 and 13 changes from glycine to alanine, cysteine, aspartic acid, arginine, serine or valine and codon 61 from glutamine to glutamic acid, histidine, lysine, leucine, proline or arginine. In KRAS, the variations at codons 12 and 13, which are the most frequent mutations associated with this protein, result in G12D and G13D substitution, respectively. Similarly, the most common mutation in HRAS is the G12V substitution. As previously mentioned, NRAS has a mutation bias at codon 61, Q61R replacement at this position being the most frequent aberration (2).

Considering that RAS mutations are all located in the homologous amino-acid region, it could be postulated that their effect on the protein function is equivalent. Nevertheless, studies have demonstrated that different substitutions in RAS proteins distinctly modify protein GTPase activity or its affinity for downstream effectors (6–8). According to these reports, different RAS mutations may result in distinct biological manifestations. As this topic is less discussed in the literature, within this review we focus on the differences among RAS proteins mutations with respect to their preferential signaling pathways, biochemistry, specific changes in cellular phenotype, mutations-specific transcriptomics, proteomics and metabolomics characteristics, as well as their individual association with patient treatment outcome and survival.

## RAS Proteins: Functional and Localization Variances

RAS proteins were initially believed to be functionally redundant due to their high homology in structure, biophysical and biochemical properties (9). Subsequently, accumulating solid experimental evidence indicated that RAS proteins differ substantially in their function in various cell types and tissues (9). For example, while, *KRAS4A-, NRAS*-, or *HRAS*-deficient mice are viable, *KRAS4B* knockout mice die during embryogenesis between days 12 and term due to liver, cardiac and hematopoietic abnormalities (10–13). These findings suggest that only *KRAS4B* may be essential during development and that there might be a redundancy in signaling among the other RAS proteins in embryogenesis. Later on, Potenza et al. modified the *KRAS* gene to encode an HRAS protein, showing that HRAS can functionally replace KRAS during embryogenesis but only under the control of KRAS promoter (6). However, these adult mice displayed dilated cardiomyopathy, indicating that KRAS has a unique role in cardiovascular homeostasis (14) and that the mortality of *KRAS*-deficient mice is likely derived from the inability of other RAS proteins to be expressed in the same subcellular compartments (9).

In relation to the protein-specific role of RAS in mouse embryogenesis, some studies have pointed out also their similar specific functions in human development. It has been shown that germline mutations in RAS proteins or in RAS regulators, such as *NF1, PTPN11*, or *SOS1*, lead to several congenital developmental disorders, such as neurofibromatosis type 1, Noonan, or Costello syndromes, respectively (9). Therefore, these data in combination with the aforementioned animal experiments indicate that normal development is regulated by a precise pattern of RAS signaling (15).

Numerous mechanistic studies from the last two decades support the notion that each RAS protein displays specific downstream signaling (16–20). The distinct protein functionalities can be attributed to different post-translational modifications occurring at the C-termini of the RAS proteins. These modifications allow RAS proteins to anchor in different subcellular membranes from where each protein can activate different signaling pathways (21). Although plasma membrane is the major location for all the RAS proteins, they have also been found in the endoplasmic reticulum, Golgi apparatus, endosomal network, and mitochondria (21). Interestingly, the level of each protein in these subcellular compartments varies according to their total abundance and between cell types. For example, Chiu et al. reported that NRAS and HRAS maintain the highest Golgi pool, followed by KRAS4A and KRAS4B, which are mainly located in the plasma membrane (18).

Early evidences from plasma membrane perturbation studies support the idea of compartmentalized RAS protein signaling. Roy et al. reported that HRAS but not KRAS4B was able to inhibit RAF/MAPK signaling pathway (16). In addition, analysis of mutant RAS proteins revealed distinctive RAF1 (CRAF) activation, with KRAS4B and KRAS4A being more potent RAF1 activators than NRAS or HRAS (17). Moreover, the protein-specific signaling leads to different outputs depending on the RAS subcellular localization (15). For example, KRAS anchored in the plasma membrane can induce cellular transformation, while its activation when located in the mitochondria triggers apoptosis (19). In the case of HRAS, Chiu et al. demonstrated that only the endoplasmic reticulum-associated form can activate the RAF1-ERK signaling pathway, leading to fibroblast transformation (18). However, HRAS Golgi-associated form seems to be unable to induce cell transformation or proliferation (20). Taken together, these data suggest that RAS protein subcellular localization modulates signaling pathway activation and its outcome.

## Phenotypical Differences Among RAS Proteins Mutations

Early studies analyzing the biochemical consequences of RAS mutations showed connections between HRAS specific mutations and cell transformation (7, 8). These reports pointed out that particular RAS mutations may modify the biochemical behavior of RAS proteins including their ability to bind GTP and GDP. Three decades later, additional differences in RAS mutations biology with respect to endpoints such as anchorage-independent growth or cell migration in many types of cancers are being continuously reported (6, 22–25), showing that RAS biological behavior is more complex than previously thought.

### Transforming Potential

Seeburg et al. were in 1984 the first to assess the transforming potential of different HRAS mutations (7) by transfecting rat fibroblasts with plasmids encoding 20 different HRAS mutant variants at codon 12, which encodes for glycine. The transforming potential of these mutants was assessed by changes in colony morphology. Rat fibroblasts expressing G12V, G12L, G12I, G12R, or G12T variants showed a fully transformed colony morphology, with cells consistently round and refractile that grew to the highest saturation densities. Interestingly, the transfection with G12K- or G12Q-mutated variants displayed low transformation, with foci induction after 2 or 3 weeks and cells with almost normal morphologies. Similarly, fibroblasts transfected with G12S, G12M, G12C, G12Y, G12F, G12W, G12H, G12D, G12E, G12A, and G12N plasmids exhibited an intermediate transformation, with cells overgrowing the monolayer but less striking changes in morphology than the most potent mutations. However, similarly to glycine, no transformation was observed with the G12P variant (7). Later, HRAS mutations at codon 61 were analyzed by Der et al. (8). NIH3T3 mouse fibroblast cells were transfected with plasmids encoding 17 different amino acids at codon 61 and the transforming potential was analyzed by foci formation (8). The transfected cells displayed different transforming potential, from very strong transforming mutants (Q61V, Q61L, Q61K, Q61A, Q61C, and Q61R) to a very weak one, Q61G, which was ~200-fold lower than Q61V. Q61H, Q61I, Q61Y, Q61M, Q61T, Q61N, Q61W, and Q61F mutants showed an intermediate spectrum between weak and strong transformation. Moreover, Q61P and HRAS WT failed to demonstrate any transformation (8). This failure is not due to the impaired expression of the mutant protein (7), but it could be explained by the fact that proline at codons 12 or 61 of HRAS displays similar biological properties as wild type (WT) HRAS (8). The overexpression of either WT HRAS or HRAS G12P or Q61P in NIH3T3 fibroblasts leads to cell transformation (8). Moreover, based on HRAS structure, proline at position 12 may cause a helix termination, resulting in a lower transforming potential (26). These early observations suggest that the transforming potential of RAS proteins also depends on the substitution that replaces the original amino acid.

Later, Smith et al. similarly compared the transforming potential of different KRAS mutations (22). NIH3T3 fibroblasts were transfected with plasmids expressing WT, G12V, G12D, G13D, and Q61H KRAS. All KRAS mutants exhibited foci formation after 21 days, however codon 12 mutations had a slightly greater transforming potential than mutations at codons 13 and 61 (G12V > G12D > G13D > Q61H) (Table 2) (22).

**Table 2:** Phenotypical differences among RAS proteins mutations.

**Characteristic/mutation**	**KRAS4A** ** G12V**	**KRAS4B** ** G12V**	**HRAS** ** G12V**	**NRAS** ** G12V**	**KRAS** ** G12A**	**KRAS** ** G12C**	**KRAS** ** G13D**	**KRAS** ** Q61L**	**KRAS** ** Q61H**	**KRAS** ** G12D**	**KRAS** ** G13C**	**KRAS** ** G12V**	**KRAS** ** G12R**	**NRAS** ** Q61R**
Transforming potential	High (17)	Low (17)	High (17)	Low (17)			High (22)		Mid (22)	Very high (22)		Very high (22)		
GTP binding						High (6)			Very high (6)		High (6)	Yes (22) High (6)		
Instrinsic GTP hydrolysis					Very slow (25)		Slow (25)	Very slow (25)	Very slow (25)	Slow (25)		Slow (25)	Very slow (25)	Very slow (24)
GAP-mediated GTP hydrolysis				Slow (25)	Very slow (25)			Slow (25)	Slow (25)	Slow (25)			Slow (25)	Very slow (24)
Anchorage-independent growth	Yes (17)	No/Yes (17)	No/Yes (17)	Yes (17)		Yes (23) Yes (6)	Yes/No (6)		Yes (6)	No (23) Yes/No (6)	Yes/No (6)	Yes (6)		
Migration	No (17)	Yes (17)Fast (30)	Minimally (17) Slow (30)	No (17)			Yes (6)			Yes (6)		Yes (6)		

*Summary of RAS mutant proteins manifestations according to different studied characteristics. GTP, guanosine-5′-triphosphate; GAP, GTPase-activating proteins*.

These intriguing data stimulated further studies in which the role of the same mutation in different RAS proteins properties has also been investigated. In that sense, Voice et al. compared transforming potential of the G12V mutation among HRAS, NRAS, KRAS4A, and KRAS4B proteins in different cell lines (17). The focus forming abilities of HRAS and KRAS4A in NIH3T3 and Rat-1 cells were ~2- to 2.5-fold higher than those of KRAS4B and NRAS. Interestingly, in RIE-1 cells, HRAS and KRAS4A transforming potentials were 8.3- and 6.3-fold higher than those of KRAS4B and NRAS (17), indicating that the differences in mutant transforming potential are also cell type-dependent (Table 2).

In addition to *in vitro* studies that have been performed to elucidate the differences among RAS mutations functional characteristics, xenograft models and genetically-engineered mouse models have been used for that purpose as well (24, 27, 28). For example, Céspedes et al. identified the tumorigenic potential of KRAS G12V and G12D mutations *in vivo* (27). Both mutations generated tumors but cells harboring the G12V mutation grew significantly faster than cells harboring the KRAS G12D mutant variant (27). A later study by Haigis et al. analyzed the transforming potential of KRAS and NRAS G12D mutant proteins expressed in the colonic epithelium of genetically-engineered mice (28). Animals harboring KRAS G12D developed widespread hyperplasia throughout the colonic epithelium, which also happened in adult mice. However, the expression of NRAS G12D mutant variant in this tissue had no effect, suggesting that KRAS might be the only RAS protein modulating the homeostasis of the colon. Interestingly, KRAS G12D mice did not develop colon cancer, indicating that the expression of this mutant variant is not sufficient to promote neoplasia (28). In addition, using a melanoma mouse model, Burd et al. reported that homozygous NRAS G12D or NRAS Q61R p16^INK4a^-deficient mice developed significantly more nevi than control mice. However, mice harboring NRAS Q61R triggered nevi formation more frequently than animals harboring NRAS G12D mutation (*p* = 0.03) (24). Moreover, the penetrance of the tumors was higher in NRAS Q61R mice than in NRAS G12D animals, results that are in accordance with the frequency of nevi formation. Nevertheless, tumor growth and histology were similar between the NRAS G12D- and the NRAS Q61R-induced tumors (24). Collectively these studies have formed a basis for the notion that the different RAS mutations display a wide variety of transforming potentials depending on various factors including the codon site, RAS protein, and cell type.

### GDP and GTP Binding

As mutations at codons 12, 13, and 61 cluster around the nucleotide-binding site, amino acids exchange at these positions may alter the interactions between RAS proteins and GTP or GDP (25). In their 1986 manuscript, Der et al. also analyzed the GDP and GTP binding affinity in WT and 17 different HRAS mutants (8). Both GDP and GTP appeared to bind WT HRAS or the HRAS Q61L mutant variant with the same affinity. In addition, the kinetics of GTP hydrolysis between WT and mutant HRAS was studied. All the analyzed mutants reduced GTP hydrolysis compared to WT HRAS, which correlates with the oncogenic activation of RAS. However, Q61L, Q61W, Q61N, Q61G, Q61P, and Q61E mutants displayed indistinguishable GTP hydrolysis, with one-eighth reduction in the rate compared to WT HRAS (8). Interestingly, these HRAS mutants have different transforming potentials, suggesting that compromised GTP hydrolysis is necessary but not sufficient for a complete RAS activation.

**Figure 1:**
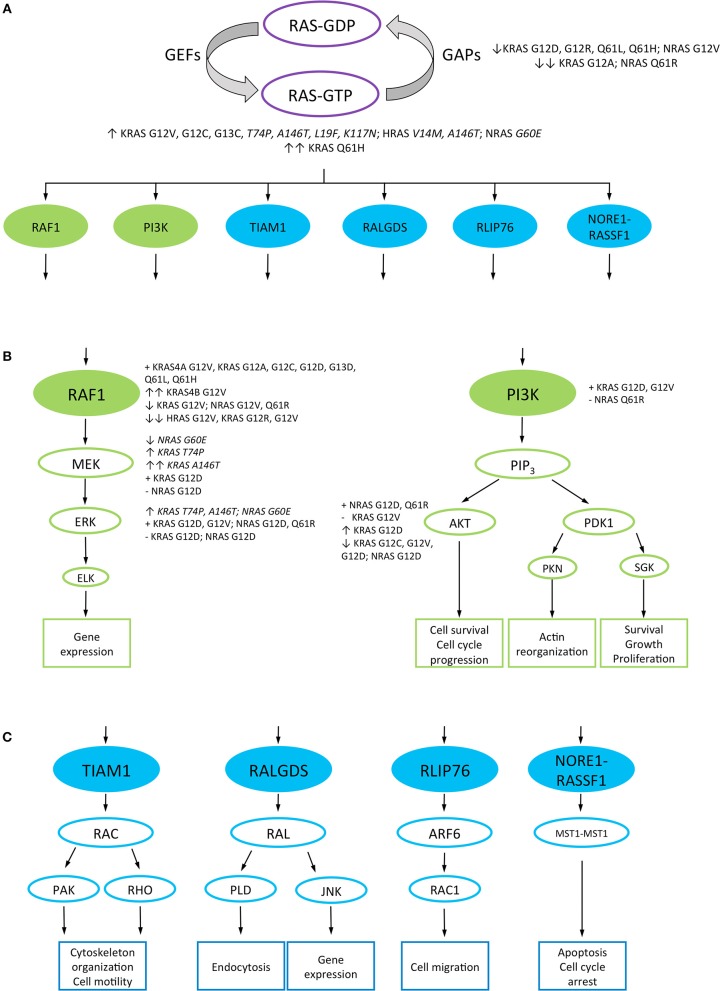
RAS downstream signaling pathways. RAS canonical and non-canonical downstream signaling pathways are represented in green and blue, respectively. RAS proteins non-canonical mutations are highlighted in italics. **(A)** RAS proteins signal between “on” and “off” conformations, given by the binding of GTP and GDP, respectively. The transition from the inactive to the active form is catalyzed by guanine nucleotide exchange factors (GEFs), while the GTPase-activating proteins (GAPs) control the inverse reaction. RAS-GTP proteins interact with different downstream effector proteins to activate several signaling pathways. RAS mutant variants which decrease GAP-mediated GTP hydrolysis and strongly bind GTP are represented. **(B)** RAS canonical downstream pathways: RAS/RAF1/MAPK and PI3K/AKT signaling pathways, and their cellular output. The ability and relative strength of different RAS mutant proteins to interact or activate effector proteins are mentioned. **(C)** A representation of the non-canonical downstream pathways of RAS and their cellular output. GEFs, guanine nucleotide exchange factors; GAPs, GTPase-activating proteins; RAF1, rapidly accelerated fibrosarcoma 1; ERK, extracellular signal-regulated kinase; MEK, mitogen-activated protein kinase; PI3K, phosphatidylinositol 3-kinase; AKT, protein kinase B; PDK, 3-phosphoinositide-dependent protein kinase; ELK, ETS Like-1 protein; PIP_3_, Phosphatidylinositol (3,4,5)-triphosphate; PKN, protein kinase N1; SGK, serum and glucocorticoid-regulated kinase; RAC, Ras-related C3 botulinum toxin substrate 1; RAL, Ras-related protein Ral; TIAM1, T-lymphoma invasion and metastasis-inducing protein 1; PAK, p21-activated kinase; RHO, Ras homologous protein; RALGDS, Ral guanine nucleotide dissociation stimulator; PLD, phospholipase D; JNK, c-Jun N-terminal kinase; RLIP76, ralA-binding protein 1; ARF6, ADP-ribosylation factor 6; RASSF1, Ras associated domain-containing protein 5; MST1, serine/threonine kinase 4; ↑, high interaction or downstream proteins activation; ↑↑, very high interaction or downstream proteins activation; ↓, low interaction or downstream proteins activation; ↓↓, very low interaction or downstream proteins activation; +, interaction or activation of the downstream effector proteins; –, inability to interact or activate the downstream effector protein.

More than 30 years later, further studies continue reporting differences in GTP binding and intrinsic or GAP-mediated GTP hydrolysis (6, 22, 25). Smith et al. detected KRAS G12V in the GTP-bound conformation, which was consistent with its high transforming potential (22). In addition, experiments in MCF10A cells transduced with different KRAS mutations revealed that WT KRAS and KRAS G12D and G13D were able to bind GTP with a similar affinity as control cells, which only express endogenous KRAS, after EGF stimulation (6). In contrast, KRAS G12C, G12V, and G13C mutants showed an increase in GTP-binding up to 2-fold and up to 5- to 6-fold in KRAS Q61H mutant compared to control cells (Figure 1A, Table 2) (6). A similar study analysing WT KRAS and KRAS mutations G12A, G12C, G12D, G12R, G12V, G13D, Q61L, and Q61H showed that the kinetics of GDP-GTP exchange were similar between all mutant proteins and WT KRAS, with the exception of KRAS G13D (25). This mutation showed a faster GDP and GTP exchange than the WT KRAS, suggesting that KRAS G13D mutant protein might be auto-activated by nucleotide exchange easier than other mutant variants. Moreover, the fast nucleotide exchange of the KRAS G13D mutant may contribute to a more aggressive biology of tumors harboring this mutation (25).

Additionally, this study also reported that while KRAS G12A, G12R, Q61H, and Q61L decreased GTP hydrolysis speed approximately by 40- to 80-fold as compared to WT KRAS, the G12C mutation had a minimal impact in this respect. Regarding this endpoint, KRAS G12D, G12V, and G13D mutant proteins displayed an intermediate effect (25). When analyzing GAP-mediated GTP hydrolysis, all KRAS mutants showed 97–99% reduction in GAP-mediated GTP hydrolysis compared to WT KRAS. In the case of KRAS G12A and Q61L, the GAP-stimulated rate was 15- to 25-fold higher than the intrinsic GTP hydrolysis rate, which may suggest that these mutants keep part of the GAP-mediated GTP hydrolysis activity (Figure 1A, Table 2) (25).

The GAP-mediated and the intrinsic nucleotide exchange were studied in tumors derived from an *in vivo* melanoma model (24). WT NRAS and NRAS G12D and Q61R mutant proteins showed similar GDP exchange rates, but differed significantly in their GTP exchange rates, with WT NRAS showing the fastest GTP exchange and NRAS Q61R the slowest. These differences were more significant when the reaction was catalyzed by GEFs. Moreover, NRAS Q61R mutant protein also showed the slowest intrinsic GTP hydrolysis (1,150- and 2,300- times slower than NRAS G12D and WT NRAS, respectively) (Table 2) (24).

These data suggest that not only intrinsic GTP hydrolysis is important for mutant RAS transformation. GAP-mediated nucleotide exchange might also have an effect on RAS mutant proteins transformation, which makes it more difficult to anticipate the transforming potential of a particular RAS mutant variant.

### Anchorage-Independent Growth

Anchorage-independent growth is the ability of transformed cells to grow in suspension or unattached to any matrix (6), an associated characteristic for tumor metastasis regulated by the RAS/RAF/MAPK signaling pathway (29). Seeburg et al. reported that with the exception of HRAS WT and HRAS G12P, all the HRAS codon 12 mutants were able to grow in soft agar (7), results paralleling their data on transforming potential of these mutant proteins.

Voice et al. also assessed the anchorage-independent growth of the G12V mutation of different RAS proteins in RIE-1 and Rat-1 cells (17). Unlike the KRAS4B G12V-harboring RIE-1 cells, same cells expressing the KRAS4A G12V mutants were able to grow in soft agar, correlating with the ability of these proteins to form foci. However, the HRAS G12V cells failed to grow in soft agar despite their ability to form foci in RIE-1 cells, whereas the NRAS G12V mutation enabled RIE-1 cells to grow in soft agar despite its little transforming activity. Interestingly, all these RAS G12V proteins enabled growth in soft agar when expressed in Rat-1 fibroblasts, although KRAS4B and NRAS showed reduced transforming potential in this cell line (Table 2) (17). This suggests that the ability to grow independently of anchorage depends on a particular cellular intrinsic milieu rather than on the RAS proteins harboring the substitution.

Later, immortalized human bronchial epithelial cells with specific shRNA knockdown of p53 mRNA expressing KRAS G12C were able to form colonies in soft agar compared to KRAS G12D- and KRAS WT-transfected cells (23), suggesting that the genetic background could also affect the phenotypical manifestation of mutant RAS variants. Moreover, Stolze et al. showed that the overexpression of KRAS G12D, G13C, and G13D in MCF10A cells yielded a very high colony number in soft agar. However, the expression of these mutants at physiological levels did not confer anchorage-independent growth (6). In the case of clones expressing KRAS Q61H, G12V, and G12C, a slight increase in colony number was observed compared to control cells expressing endogenous KRAS, which also correlated with the highest GTP-bound levels reported in the same study (Table 2) (6). Collectively, these results suggest that some RAS mutant proteins might have the ability to grow independent of anchorage, which may depend on cell type and genetic background.

### Migration

Cell migration is controlled by several RAS downstream pathways, such as the RAS/RAF/MAPK pathway (29). As this process involves cancer cells local invasion and metastasis (6), several studies analyzed the migration abilities of distinct RAS mutant proteins (6, 17, 30). Voice et al. reported that the KRAS4B G12V variant could accelerate COS-7 cells migration while HRAS G12V had a minimal effect, compared with cells transfected with GFP alone. However, KRAS4A G12V- and NRAS G12V-expressing cells were unable to migrate, even at higher expression levels (17). A later study by Walsh et al. (30) showed that KRAS4B G12V-transfected REF-52 cells migrated at the speed of 18 μm/h, while the HRAS G12V cells at 12 μm/h (30). In addition, Stolze et al. reported that the overexpression of KRAS G12D, G12V, and G13D enabled MCF10A cells migration (6). Similarly to KRAS-overexpressing mutant proteins, control cells were able to migrate after EGF addition (Table 2). However, none of the studied mutations expressed at physiological levels increased migration abilities compared to WT KRAS or control cells, which expressed endogenous KRAS (6). Therefore, these results contrast with previous studies (17, 30) as only the overexpression of KRAS mutant variants leads to cell migration.

Animal model studies also evaluated metastatic capacities of tumors harboring KRAS mutations (31, 32). A recent *in vivo* study by Tang et al. analyzed tumor formation and their metastatic capacity in KRAS G12D p53^−/−^ mice (31). As compared to KRAS^WT/WT^ p53^−/−^ and KRAS^WT/WT^ p53^+/+^ (wild type) mice, animals harboring both KRAS G12D and p53^−/−^ alterations developed tumors with 100% penetrance and their size increased over time. Moreover, tumors from KRAS G12D p53^−/−^ mice were able to metastasize to the liver, spleen and kidney whereas tumors formed in KRAS^WT/WT^ p53^−/−^ and WT animals were not (31). Previously, Whipple et al. studied the involvement of the heparin sulfate proteoglycan Glypican-1 (GPC1) in KRAS G12D-driven mouse model of pancreatic cancer (32). At 65 days of age, 14 of 14 animals harboring wild type GPC1 developed large pancreatic tumors that invaded the surrounding organs, whereas 16 of the 20 GPC1^−/−^ mice developed smaller and non-invasive tumors (32). Moreover, four primary cancer cell lines were derived from tumors developed in GPC1^+/+^ (F1015 and F1048) and GPC1^−/−^ (J444 and J1032) mice. These cell lines formed tumors in GPC1^+/+^ nude mice. However, *in vitro* studies revealed that J444 and J1032 cells exhibited decreased invasion capacities in response to FGF-2 compared to F1015 and F1048 primary cancer cells (32). To determine whether the loss of GPC1 was also involved in a reduction of invasion *in vivo*, tumor fragments from GPC1^+/+^ and GPC1^−/−^ mice were implanted into the pancreas of athymic GPC1^+/+^ and GPC1^−/−^ animals. Two weeks after the implantation, only 2 out of 14 GPC1^−/−^ mice developed metastasis in the mesentery, while 9 out of 15 GPC1^+/+^ mice developed several (over 100 per animal) mesenteric metastases and three of them also showed multiple renal metastases (32). Therefore, these two studies suggest that not only KRAS G12D-expressing tumors are able to migrate and metastasize *in vivo*, but also other RAS mutant proteins may have the capacity to invade the surrounding tissues, as reported by *in vitro* studies (6, 17, 30).

All together these findings strongly indicate that point mutations at codons 12, 13, and 61 of RAS display different phenotypical characteristics compared to WT RAS. Depending on the RAS isoform and the amino acid substitution, RAS mutant proteins differ in their transforming ability, GTP binding, anchorage-independent growth and migration capacities. But these results also suggest that RAS mutations show a different biological behavior depending on the cell type where they are expressed, adding complexity to our understanding of RAS biology.

## Mutant RAS Proteins Differ in Their Biochemical Signaling

Wild type RAS proteins are able to activate different signaling pathways depending on particular cell type, tissue and their subcellular localization (21). As codon 12, 13, and 61 mutations are located around the nucleotide-binding site, it has been suggested that the nucleotide exchange may alter the affinity of mutant RAS proteins for downstream effectors proteins (17, 25).

### Activation of the RAF1/MAPK Pathway

The RAF1 serine/threonine kinase is one of the best characterized RAS effector proteins, located directly downstream of RAS in the MAPK pathway (25). Considering that point mutations at codons 12 and 61 of HRAS differ in their phenotypical properties as previously reported (7, 8), Voice et al. hypothesized that mutant RAS proteins might activate RAF1 differentially (17). The co-transfection of WT RAF1 and G12V HRAS, NRAS, KRAS4A, and KRAS4B in COS-1 cells confirmed that RAS proteins differ in their ability to activate RAF1. KRAS4B activated RAF1 8.4-, 4.4-, and 2.3-fold better than HRAS, NRAS, and KRAS4A, respectively, proposing the following hierarchy in RAF1 activation by these RAS proteins: KRAS4B > KRAS4A >>> NRAS > HRAS (Figure 1B) (17). Later, Hunter et al. analyzed the affinity of different KRAS mutants for the RAS-binding domain (RBD) of RAF1 (25). KRAS G12A, G12C, G13D, Q61L, and Q61H showed 1.2- to 2.3-fold decrease in relative affinity compared to WT KRAS and KRAS G12D, G12R and G12V displayed even more pronounced decrease in affinity for RAF1 (4.8-, 6.2-, and 7.3-fold, respectively) (Figure 1B, Table 3) (25). These results contrast with those reported by Voice et al. (17) where KRAS G12V showed a high activation of RAF1. However, these differences could be related to the method used in each study to detect RAF1 activation.

**Table 3:** Interaction and activation of different RAS proteins downstream effectors.

**Characteristic/mutation**	**KRAS4A** ** G12V**	**KRAS4B** ** G12V**	**HRAS** ** G12V**	**NRAS** ** G12V**	**KRAS** ** G12A**	**KRAS** ** G12C**	**KRAS** ** G13D**	**KRAS** ** Q61L**	**KRAS** ** Q61H**	**KRAS** ** G12D**	**KRAS** ** G12R**	**KRAS** ** G12V**	**NRAS** ** G12D**	**NRAS** ** Q61R**
RAF1 interaction	High (17)	Very high (17)	Very low (17)	Low (17)	High (25)	High (25)	High (25)	High (25)	High (25)	Low (25) – (27)	Very low (25)	Very low (25) + (27)	High (24)	Low (24)
ERK activation			Strong (33)							–(27) + (28)		Low (33) + (27)	– (28) + (24)	+ (24)
MEK activation										+ (28)			– (28)	
PI3K interaction										+ (27)		+ (27)		Low (24)
AKT activation						Low (23)				Strong (27) Decreased (28)		Low (23) (27)	Decreased (28) + (24)	+ (24)
70S6K activation						Strong (23)				Strong (23)				
RPS6 activation							High (6)							
RAC interaction		Strong (30)	Low (30)							– (28)			– (28)	
RAL interaction						+ (23)				– (23, 28)			– (28)	

*Summary of the activation of downstream signaling pathways by RAS mutant proteins. Proteins activation was assessed by phosphorylation at different residues. +, interaction or activation of the effector; –, inability to interact or activate the downstream effector protein; RAF1, rapidly accelerated fibrosarcoma 1; ERK, extracellular signal-regulated kinase; MEK, mitogen-activated protein kinase; PI3K, phosphatidylinositol 3-kinase; AKT, protein kinase B; 70S6K, ribosomal protein S6 kinase 1; RPS6, ribosomal protein S6; RAC, Ras-related C3 botulinum toxin substrate 1; RAL, Ras-related protein Ral*.

Other works analyzed the activation of the MAPK pathway by assessing ERK activation through its phosphorylation status (6, 33). For example, transduction of primary rat hepatocytes with HRAS G12V, but not with KRAS G12V, showed a strong activation of ERK2 independently of EGF stimulation (33), revealing that different RAS proteins harboring the same mutation activate downstream signaling pathways differently. On the other hand, a more recent study by Stolze et al. reported no differences in ERK phosphorylation levels in MFC10A cells expressing KRAS G12D, G12V, G12C, G13D, G13C, and Q61H at low levels compared to WT KRAS or control cells, expressing endogenous KRAS (Figure 1B, Table 3) (6). These data suggest that not only the cell type but also the level of expression may influence the pattern and intensity of RAS mutations signaling pathways activation.

Mutant RAS signaling differences have also been identified in tumors derived from animals. Céspedes et al. reported that mouse tumors expressing KRAS G12V, but not G12D, were able to interact with RAF1 and showed a high phosphorylation of ERK (27). Interestingly, Haigis et al. reported different results concerning KRAS G12D (28), where KRAS G12D but not NRAS G12D could activate MEK and ERK in colonic epithelium of genetically engineered mice (Figure 1B). However, the activation of both KRAS G12D and NRAS G12D proteins at the same time only appeared in the differentiated cells at the top of the colonic crypt and not in the undifferentiated cells at the bottom of the crypt, suggesting that the exact activation pattern of ERK depends on the cell type (28). Recently, a study by Burd et al. revealed that NRAS Q61R bound RAF1 with lower affinity than WT NRAS or NRAS G12D in melanoma. However, both NRAS Q61R and G12D mutant proteins activated ERK at variable levels (Figure 1B, Table 3) (24), suggesting that the activation of MAPK pathway in melanoma is codon-independent.

### PI3K/AKT/mTOR Pathway Activation

RAS proteins also trigger the activation of the PI3K/AKT/mTOR pathway to promote cell survival by activating survival factors and inhibiting apoptotic proteins (5). Therefore, different *in vitro* (6, 23) and *in vivo* (24, 27, 28) studies also assessed the activation of this pathway by the interaction of various RAS mutated variants with PI3K and different downstream proteins phosphorylation, such as AKT, 4EBP, or RPS6. The comparison of KRAS G12C and G12V with WT KRAS in a panel of 67 non-small cell lung cancer cell lines showed that these mutations decreased AKT activation compared to WT KRAS (Figure 1B) (23). Despite this low activation of AKT, cells expressing KRAS G12C or G12V showed the same phosphorylation levels of 70S6K and 4EBP proteins compared to WT KRAS in the absence of serum whereas the addition of serum to the media enabled KRAS G12C and G12V to strongly phosphorylate 70S6K compared to WT KRAS (Figure 1B, Table 3) (23). Later on, Stolze et al. reported that KRAS G12D, G12V, G12C, G13D, G13C, and Q61H expressed at low levels in MCF10A cells did not show higher phosphorylation of PDK1 and AKT compared to WT KRAS or control cells which only express endogenous KRAS (6). Nevertheless, both KRAS G13D low expression as well as overexpression were associated with a high RPS6 phosphorylation upon EGF (Table 3), indicating that this mutant enabled mTOR pathway activation but seemingly not through the PI3K/AKT pathway (6). Interestingly, the results reported for KRAS G12D in this study are inconsistent with those obtained by Ihle et al. (23), suggesting that the cell type and/or the genetic background may alter the activation of the downstream signaling pathways.

In mouse xenograft tumors, Céspedes et al. showed that both KRAS G12V and G12D mutants were able to interact directly with PI3K (27). However, KRAS G12V was unable to activate AKT despite its interaction with PI3K whereas KRAS G12D strongly activated AKT (27). Contrary, KRAS G12D as well as NRAS G12D expressed in the mouse colonic epithelium showed a decrease in AKT phosphorylation compared to WT animals (Table 3) (28), proposing once again that cell type may alter the downstream signaling pathways activation. In addition, studies employing a mouse model of melanoma revealed that NRAS Q61R binds PI3K with lower affinity than WT NRAS or NRAS G12D while NRAS G12D and Q61R activate AKT at variable levels (Figure 1B, Table 3) (24), indicating that the activation of the PI3K pathway in melanoma is codon-independent.

### Other Effectors Activation

RAS proteins can also interact and activate effectors that do not belong to the MAPK and the PI3K canonical cascades. For example, RAC, a subfamily of small GTPases of the RHO family, can interact with RAS via the RacGEF called Tiam1. The RAS/RAC signaling pathway controls several cellular functions through the regulation of actin cytoskeleton, including cell morphology, locomotion, and polarity (34). Another RAS downstream effector subfamily is the RAL group of proteins, which are involved in membrane trafficking, proliferation, survival and metastasis in many types of cancer (35).

Walsh and Bar-Sagi studied the differential activation of RAC in COS-1 cells transduced with KRAS4B G12V and HRAS G12V (30). Cells expressing KRAS4B G12V activated RAC more effectively than HRAS G12V-transduced cells (30). Moreover, *in vivo* studies also analyzed the modulation of RAC (28). The expression of either KRAS or NRAS G12D in the mouse colonic epithelium did not promote RAC modulation. In addition, this study revealed that KRAS and NRAS G12D were unable to modulate RAL activation (Table 3), indicating the limited signal activation of these mutants *in vivo* (28). These results are consistent with a later *in vitro* study (23), in which WT KRAS and KRAS G12C, but not KRAS G12D, where able to activate RALA and RALB effector proteins (Table 3) (23).

Furthermore, Stolze et al. analyzed whether any of the KRAS codon 12, 13, and 61 mutations included in the study was able to increase the activation of EGFR and p53 (6). Only KRAS G13D ectopically expressed in MCF10A breast cancer cells promoted an increase in total and phosphorylated EGFR. Moreover, KRAS G13D stimulated a strong phosphorylation of p53 at serine 15, a site known to be phosphorylated by the master DNA damage response kinase ATM, which suggests that particularly the KRAS G13D mutant might induce a DNA damage response under replicative stress (6). As other KRAS mutants did not show the activation of EGFR and p53, these authors suggest that this activation could be the biological explanation of the favorable clinical outcome of colorectal cancer patients harboring KRAS G13D mutation treated with anti-EGFR therapy compared to patients with KRAS codon 12 mutations (6).

### Signaling Pathway Activation and Outcome

Several studies assessing the impact of RAS mutations on cell behavior correlated the signaling pathways activated by a specific mutation with a particular outcome such as cell death or cell cycle redistribution (27, 28, 30, 33). Joneson and Bar-Sagi reported that overexpression of HRAS G12V induced apoptosis in a panel of primary and immortalized cells (36). However, the co-transfection of REF-52 fibroblasts with HRAS G12V and activated RAC blocked HRAS G12V-induced apoptosis, indicating that RAC signaling pathway is sufficient to antagonize RAS proapoptotic signals (36). As KRAS4B G12V and HRAS G12V differentially activate the RAC signaling pathway (30), Walsh and Bar-Sagi hypothesized that these mutant variants may differ in their ability to induce apoptosis (30). The overexpression of HRAS G12V in REF-52 fibroblasts induced apoptosis in 38% of the cells whereas the overexpression of KRAS4B G12V had no effect on cell viability, results that are consistent with the RAC activation levels reported in this work for each mutant (30).

Céspedes et al. described that expression of KRAS G12V in xenograft tumors enhanced Retinoblastoma (Rb) protein phosphorylation and was accompanied by an increase in cyclin B1 expression. This could be related to the high proliferation rate of these tumors and their fast G1/S and G2/M transitions (27). However, no differences in the level of procaspase 3 or 9 proteolysis were detected between KRAS G12V and G12D tumors, leading to a similar activation of apoptosis (27). In a later study, Haigis et al. exposed genetically engineered mice to 2.5% dextran sodium sulfate (DSS). Mice expressing WT KRAS or KRAS G12D in the colonic epithelium were sensitive to DSS-induced apoptosis in this tissue, whereas in mice expressing NRAS G12D little or no apoptotic effect was observed. However, NRAS G12D mice were sensitive to irradiation-induced apoptosis in the colonic epithelium, indicating that the effect of this mutation might depend on the apoptotic stimuli and the activated cell death pathway (28).

Along similar lines of investigations, Rosseland et al. studied proliferation of primary rat hepatocytes transfected with HRAS G12V or KRAS G12V (33). Compared to control cells expressing the yellow fluorescent protein, the proliferation rate of HRAS G12V, but not of KRAS G12V, was increased after EGF stimulation (33). In addition, an earlier study by Oberhammer et al. reported that TGF-βI increased the incidence of apoptosis in hepatocytes by 5-fold, suggesting that TGF-βI is involved in the initiation of apoptosis in the liver (37). Based on these results, Rosseland et al. tested whether HRAS G12V and KRAS G12V were able to induce apoptosis in rat hepatocytes after TGF-βI stimulation (33). Hepatocytes expressing HRAS G12V or KRAS G12V had reduced apoptosis compared to untransfected control cells, demonstrating that both RAS mutant proteins have a pro-survival effect (33). To further investigate the signaling pathways involved in this phenomenon, PI3K and ERK pathways were blocked with different inhibitors. In untransfected control cells, apoptosis was only slightly increased after ERK pathway inhibition while PI3K inhibition strongly increased apoptosis, indicating that both ERK and PI3K pathways are involved in survival of primary hepatocytes (33). In HRAS G12V-transfected hepatocytes, the inhibition of ERK or PI3K pathways did not reduce apoptosis after TGF-βI stimulation. However, in KRAS G12V-transfected cells, only the inhibition of PI3K pathway showed an increase in hepatocytes apoptosis (33). This suggests that apoptosis is triggered through different pathways depending on the RAS isoform and mutation.

Taken together, a single amino acid change at codons 12, 13, or 61 of RAS alters the interaction of these proteins with the downstream effectors. Depending on the RAS protein and the amino acid substitution, RAS mutants activate differently the canonical and non-canonical downstream signaling pathways *in vitro* and *in vivo*. Moreover, the amino acid substitutions have been correlated with a particular outcome, such as proliferation or cell death and hence these observations should be further exploited and considered for the choice of treatment of patients.

## RAS Mutated Variants Differ in Their Transcriptomic, Proteomic and Metabolomic Profiles

Protein and metabolic stress are two recognized hallmarks of cancer in which different cellular signaling pathways are altered to confer an advantage to cancer cells and sustain their growth and proliferation (38). To get insights into global cellular networks that underlie various RAS mutated variants, various works have been assessing the transcriptomic, proteomic/phosphoproteomic and metabolic profile of RAS mutant variants to possibly associate and understand the basis of their phenotypic disparities (6, 39–41).

### Transcriptomics

Roberts et al. analyzed whether the expression pattern of 2,100 genes involved in cancer progression differ between KRAS G12V- and HRAS G12V-expressing Caco-2 colorectal adenocarcinoma cells and found 71 differentially regulated genes (42). KRAS G12V significantly up-regulated the expression of genes in the cytokine/chemokine family, for example *CD40L, CD27L, CD30L*, and *TRAF-5* and regulated processes related to immune response, development, nucleotide excision repair, cell proliferation, transcription and cytokine signaling (42). HRAS G12V-expressing cells up-regulated vimentin and down-regulated villin and fibronectin, correlating with the main biological processes controlled by HRAS G12V such as cell-matrix and cell-cell adhesion, protein biosynthesis, integrin-mediated signaling, cell motility and cell cycle checkpoint control, most of them involved in the epithelial-mesenchymal transition (42). In addition, this work assessed changes in the transcriptome profile *in vivo*, revealing 26 genes differentially expressed between KRAS G12V and HRAS G12V tumors. Up-regulation of Notch signaling, cell motility or microtubule cytoskeleton were detected in KRAS G12V whereas genes involved in cell adhesion and motility were deregulated and those involved in organogenesis/angiogenesis and cytokinesis processes down-regulated in HRAS G12V tumors (42). Later, to provide insights into the differential response of KRAS G12D and KRAS G13D mutant variants to anti-EGFR therapy, Stolze et al. compared the gene expression of these mutants and WT KRAS (6). The analysis of 2,487 genes demonstrated that WT KRAS and control MCF10A cells, expressing endogenous KRAS only, had a similar expression profile, while KRAS G12D- and G13D-expressing cells showed a different one, clustering them separately from each other and from the WT KRAS and control cells (6). Moreover, this work identified, 11,207 and 1,011 genes significantly up- and down-regulated, respectively, in KRAS G13D compared to KRAS G12D-expressing cells (6). The analysis of the top 300 up- and down-regulated genes in both mutants and their comparison to luminal and basal/mesenchymal breast cancer gene expression profiles reported previously (43, 44), associated KRAS G13D with the basal/mesenchymal and KRAS G12D with the luminal breast cancer subtype. Thus, KRAS G13D mutant variant highly expressed genes such as those encoding for integrins, collagens, and proteases, compared to KRAS G12D (6). Furthermore, Stolze et al. were able to identify mutation-specific signaling networks: 87 out of 300 top up-regulated genes were included in a cluster associated with cytokine-induced cell migration. In this cluster, the top up-regulated cytokines were *CXL1, IL1B*, and *IL8*, which showed > 10-fold increase in their transcription in KRAS G13D-expressing MCF10A cells compared to KRAS G12D-expressing cells (6).

Recently, KRAS G13D transcriptomic profile has been reported also by Charitou et al. for the isogenic HKe3 colorectal cancer cell line expressing WT KRAS or KRAS G13D (40). More than 6,000 genes were identified to be differentially expressed between WT KRAS- and KRAS G13D-expressing cells. Pathway analysis of up-regulated genes revealed that ribosome biogenesis, mRNA translation, regulation of gene expression and metabolism were among the most significantly enriched processes in cells expressing KRAS G13D (40). Metabolic stress is a recognized hallmark of cancer. To respond to the high energetic demand, cancer cells increase ribosome biogenesis to translate mRNAs into proteins in response to their high metabolic rate. In this respect, some metabolic pathways were also up-regulated in KRAS G13D-expressing cells compared to WT KRAS-expressing cells. These pathways include glycolysis/gluconeogenesis, steroid biosynthesis and glycine, serine and threonine metabolism (40). The steroid biosynthesis pathway has cholesterol as its final product. It has been reported that oncogene-transformed cells require high levels of cholesterol to support their rapid growth (45, 46). These results suggest that KRAS G13D-expressing cells might have a higher metabolic rate compared to cells expressing other KRAS mutant variants. Moreover, among the down-regulated genes in the KRAS G13D-expressing HKe3 cells, the most enriched pathways were the type I interferon signaling pathway and the antigen processing and presentation pathway, which may help cancer cells to evade the host immune response (40).

Jiang et al. analyzed the differences in both protein and microRNA (miRNA) gene expression of NRAS Q61K-, Q61L-, and Q61R-driven melanomas compared to those expressing WT NRAS (47). One thousand one hundred fifty protein-coding genes were significantly differentially expressed, with 469 and 681 up- and down-regulated, respectively, in NRAS Q61K, Q61L, and Q61R samples compared to the WT NRAS samples. In the case of miRNAs, the expression of 49 miRNAs was altered, with 26 and 23 up- and down-regulated, respectively (47). Moreover, this work identified pathways associated with these deregulated genes and miRNAs; the most significant ones in both deregulated genes and miRNAs were the MAPK signaling pathway, followed by the PI3K/AKT and the CDK/4/6/Rb pathways (47). The MAPK pathway is altered in most melanomas, while PI3K/AKT pathway is involved in melanoma initiation and its therapeutic resistance (48). In addition, CDK4 is a regulator of the G1/S cell cycle checkpoint, and its targeting using Palbociclib has demonstrated antitumor activity in melanoma (47). Other signaling pathways were also enriched in NRAS-mutated melanoma, including pathways involved in calcium, TGF-β, and WNT signaling, actin cytoskeleton, focal adhesion and axon guidance, suggesting them as novel candidate pathways for melanoma treatment (47).

### Proteomics and Phosphoproteomics

Hammond et al. investigated proteomics and phosphoproteomics signatures of isogenic SW48 colorectal cancer cell lines expressing either WT KRAS or KRAS G12D, G12V, or G13D variants (49). Hierarchical clustering of proteomic and phosphoproteomic data revealed that KRAS G12D- and G12V-expressing cells had similar signatures, but these were different from KRAS G13D-expressing cells. KRAS G13D showed more proteins and phosphopeptides up-regulated (around 50% compared to WT KRAS) than KRAS G12D-expressing cells (<10% compared to WT KRAS) (49). These findings suggest that specific mutated codons define different proteomic and phosphoproteomic signatures. In addition, same authors assessed in this work proteins and phosphoproteins differentially expressed in KRAS G12D and G13D to determine whether a codon-specific signature could be found (49). The analysis of the proteomes revealed that the expression of mitochondrial proteins involved in oxidative phosphorylation was decreased in KRAS G13D-expressing SW48 cells compared to KRAS G12D-expressing cells. Moreover, KRAS G13D showed a decrease in 5 members of the cytochrome bc1 complex (complex III) and succinate dehydrogenase of complex II of the mitochondrial respiratory chain. In contrast, the expression of aldehyde dehydrogenase (ALDH3A1) was increased in KRAS G13D-expressing cells and decreased in KRAS G12D-expressing SW48 cells (49). Regarding the phosphoproteomic data, MET Thr995 and Caveolin-1 Ser37 sites exhibited >10-fold increased abundance in KRAS G12D as compared to KRAS G13D, explained by an increase in protein expression, while BRAF Ser729 phosphorylation was decreased in KRAS G12D vs, G13D-expressing cells. These results were further confirmed in a panel of 275 lung, pancreas and colon cancer cell lines harboring KRAS codon 12 and 13 mutations or WT KRAS (49). In addition, this work identified the doublecortin-like kinase 1 (DCLK1) protein levels to be at least 8-fold up-regulated in KRAS G12D-expressing SW48 cells compared to WT KRAS-expressing cells. However, qPCR analysis revealed that the increased levels of DCLK1 are due to transcriptional up-regulation, and this increase in the mRNA level is reversed upon KRAS knockdown, indicating that KRAS directly regulates DCLK1 expression (49). DCLK1 is frequently overexpressed in colorectal cancer (50) and has been identified as a colorectal cancer stem cell specific marker, whose depletion promotes polyps regression (51). Moreover, a KRAS synthetic lethal screening previously identified the related kinase DCLK2 as a hit in the colorectal DLD-1 cell line (52), suggesting DCLK1 as a potential target for combination therapy in the context of KRAS-mutated colorectal cancer.

Concerning HRAS mutant variants, Doll et al. profiled the proteomic and phosphoproteomic changes in HRAS G12V-transformed normal human astrocytes (53). Two hundred and seventy-eight phosphosites in 154 proteins and 245 phosphorylation sites in 160 proteins were up- and down-regulated, respectively, in WT HRAS- vs. HRAS G12V-expressing cells. The analysis of these up-regulated phosphosites revealed that the MAPK, PI3K/AKT and mTOR pathways were significantly up-regulated in HRAS G12V-expressing astrocytes as compared to WT HRAS cells (53). In the MAPK pathway, Sprouty 4, whose expression is induced by this pathway, showed 10-fold upregulation at protein level. Regarding PI3K/AKT, the Niban protein (FAM129A), which regulates the phosphorylation of the transcription factor EIF2A, showed 2-fold upregulation in HRAS G12V-expressing cells. Moreover, the phosphorylation of RPTOR on Ser863 showed a 2.6-fold upregulation (53). This phosphosite is involved in mTORC1 activation, whose signaling activates different transcription factors involved in transcription of cell proliferation and survival proteins (54). This work also identified other deregulated proteins downstream of HRAS. For example, six of the 13 RAL direct downstream effectors of RAS involved in endocytosis and gene expression (Figure 1), including RALA and RALB, showed 2-fold or higher upregulation at protein level (53). Collectively, these results indicate that HRAS G12V mainly activates the canonical downstream pathways of RAS, triggering changes in gene expression that facilitate cancer cells proliferation and survival.

Interestingly, Santra et al. recently reported differences in HRAS G12V signaling according to its subcellular localization in HeLa cells (41). Three hundred and ninety-seven proteins that interact with HRAS G12V were identified across plasma membrane (PM), lipid rafts (LR), endoplasmic reticulum (ER) and Golgi apparatus (GA), out of which 341 were new interactors. Only 5% of the interactors were identified in all subcellular localizations, whereas ~53% were specific for one of the localizations (41). The pathway enrichment analysis revealed that HRAS G12V not only regulates receptor tyrosine kinase (RTK) signaling, but also biosynthesis and metabolic pathways mainly from the ER, while immune signaling is triggered from the GA. Additionally, lipid biosynthesis pathways were also enriched (41), a finding which might be related to changes in cellular metabolism. This work also assessed changes in the phosphoproteome of HRAS G12V-expressing cells according to its subcellular localization (41). One thousand four hundred sixty-one phosphosites in 1,078 proteins were differentially phosphorylated, with 74% of the phosphosites activated at LR and PM (41). The analysis of the enriched pathways showed that HRAS G12V-expressing cells regulate RTK signaling and other signaling pathways, such as WNT, MAPK, or insulin signaling pathways (41). The results of this work confirm previously described findings that apart of subcellular localization-specific differences in RAS WT proteins signaling (20, 21), also RAS mutant variants may signal differently depending on the particular cellular membrane where they are anchored, thus increasing the complexity of RAS signaling.

With respect to NRAS mutant variants, Posch et al. analyzed the differences in the phosphoproteomic profile of primary human melanocytes (PHMs) transfected with WT NRAS and NRAS G12V or Q61L (55). One hundred and sixty-three phosphosites in 132 proteins were differentially phosphorylated between NRAS G12V and WT NRAS, with 83 and 80 phosphosites up- and down-regulated, respectively. PHMs expressing NRAS Q61L showed 202 phosphosites in 150 proteins differentially regulated compared to PHMs expressing WT NRAS, with 73 and 129 phosphosites up- and down-regulated, respectively. Posch et al. also identified 126 proteins and 163 phosphosites 2-fold differentially regulated between NRAS G12V- and NRAS Q61L-expressing cells (55), indicating that both NRAS G12V and Q61L have different phosphoproteomic profiles. Moreover, this work assessed the enriched canonical pathways regulated by each NRAS mutant. Whereas, NRAS Q61L-expressing cells showed an overrepresentation of phosphopeptides related to the MAPK signaling pathway, NRAS G12V had an enrichment of the “14-3-3-mediated”- pathway, which is related to the PI3K/AKT signaling pathway due to the modulation of PI3K signaling by 14-3-3 protein (55). To confirm these results, changes in the phosphorylation level of AKT, RPS6, MEK and ERK were determined. While NRAS G12V-expressing cells showed an increase in AKT and RPS6 phosphorylation levels, NRAS Q61L-expressing cells showed an increase in MEK and ERK phosphorylation levels (55). These data suggest that NRAS G12V preferentially signals through the PI3K/AKT pathway while NRAS Q61L activates the MAPK pathway. In addition, Posch et al. determined kinases differentially expressed between NRAS G12V and Q61L cells. PHMs expressing NRAS G12V showed an overrepresentation of phosphosites associated with the PIM2 kinase and other kinases related to the PI3K/AKT signaling pathway, which correlates with the pathway enrichment reported in this work, while NRAS Q61L-expressing cells showed enriched CK2α kinase-related sites (55). This *in silico* prediction was later confirmed by analyzing clinical samples of NRAS mutant melanoma. Sixteen out of 18 NRAS Q61 mutated melanomas and one out of 2 NRAS G12 mutant melanomas showed a positive expression for CK2α, with higher expression levels in the NRAS Q61 mutant samples (55). Moreover, the TCGA data set for skin cutaneous melanoma was analyzed to determine whether CK2α was differentially expressed between NRAS Q61 and NRAS G12 mutant melanomas. The comparison of CK2α mRNA levels between both NRAS Q61 and G12 mutant melanomas showed a higher expression of CK2α in NRAS Q61 mutant samples (55), confirming thus the *in silico* prediction. CK2α is a constitutively active serine/threonine protein kinase involved in many cellular processes, such as cell growth, proliferation, and survival (56). Recently, its role in antitumor drug resistance has been reviewed, pointing to the modulation of PI3K/AKT, β-catenin and other signaling pathways directly involved in drug resistance by CK2α. Moreover, the available CK2α inhibitors (56) are under evaluation to determine whether this kinase is a potential target in cancer treatment.

### Metabolomics

Brunelli et al. characterized the metabolic profile of the isogenic NCI-H1299 NSCLC cell line overexpressing WT KRAS or KRAS G12C, G12D, or G12V (38). The majority of metabolites identified were common to all three KRAS-mutated lines (G12C, G12D, and G12V), although these mutants harbored 74, 58, and 48 unique metabolites, respectively, compared to WT (38). Moreover, the deregulated metabolites between WT and mutant KRAS variants were classified into biochemical groups. The two most abundant classes for KRAS G12C, G12D, and G12V were glycerophospholipids and amino acids. KRAS G12C and G12D mainly affected phosphatidylcholines (PC) and phosphatidylinositols (PI), whereas KRAS G12V influenced PI and phosphatidylserine (38). In addition, the report by Brunelli et al. provided further insights over the biology of the deregulated metabolites. KRAS G12C, G12D, and G12V variants showed an increase of metabolites related to protein biosynthesis, glutathione, glutamate metabolism and ammonia recycling (38). Regarding the protein synthesis pathway, all these mutants displayed greater levels of tryptophan and lower levels of the rest of the amino acids compared to WT KRAS, with the exception of the high amount of phenylalanine found in KRAS G12D-expressing cells (38). Moreover, KRAS G12C, G12D, and G12V had lower levels of glutamate, glutamine, asparagine and proline, amino acids interconnected in the glutamate synthase cycle, and lower levels of NAD^+^, an essential coenzyme involved in many cellular metabolic pathways (38). Glutamate and glutamine are two amino acids involved in glutaminolysis, one of the central cellular pathways that fuel cancer cells growth and proliferation, which also support the production of antioxidant molecules such as glutathione. Considering the low levels of glutamine reported in this work (38), Brunelli et al. studied glutathione cellular levels. All analyzed KRAS mutant variants showed low levels of reduced glutathione (GSH) and pyroglutamic acid, both involved in glutathione metabolism. However, the GSH level was slightly higher in KRAS G12C than in KRAS G12D and G12V, but not different from WT KRAS (38).

Following on these results (38), the group of Roberta Pastorelli continued studying the metabolic profile of KRAS G12C, as it is the most representative KRAS mutation in NSCLC patients. In this work, the NCI-H1299 NSCLC cell line expressing WT or KRAS G12C and xenograft tumors generated from this cell line were analyzed (39). Brunelli et al. identified 26 and 23 deregulated metabolites *in vitro* and *in vivo*, respectively, between WT KRAS and KRAS G12C. The enriched pathway analysis of these deregulated metabolites showed that KRAS G12C alters the same metabolic pathways *in vitro* and *in vivo*, including pathways involved in protein biosynthesis, ammonia recycling, and urea cycle (39). Focusing on the deregulated metabolites whose abundance changed significantly *in vitro* and *in vivo* between WT KRAS and KRAS G12C, 11 and 16 metabolites were significantly altered, respectively. Moreover, in both *in vitro* and *in vivo* models, KRAS G12C decreased the levels of glutamine and glutamate, two amino acids involved in nitrogen balance maintenance, supporting the central role of glutaminolysis and nitrogen anabolism to provide energy for cancer cell growth and proliferation. This indicates that cells expressing the KRAS G12C variant use glutaminolysis as a source of energy (39). In addition, KRAS G12C mutation induced a significant increase in the levels of carnitine, acetyl-carnitine and butyryl-carnitine, which are involved in the oxidation of fatty acids (39). This increase could be associated with the mitochondrial fatty acid beta oxidation to respond to the increasing energy demand triggered by KRAS G12C to fuel cell or tumor growth and proliferation (39). Moreover, the same group previously reported that KRAS G12C-expressing cells mainly affected PC and PI (38), showing later a down-regulation of some PC species *in vitro* but not *in vivo* compared to WT KRAS. These changes have been reported to be an important source of second messengers that could play a role in the MAPK and PI3K/AKT signaling pathways that are commonly altered in cancer (57).

In addition to the transcriptomic profile, Charitou et al. also assessed the metabolic differences between WT KRAS- and KRAS G13D-expressing HKe3 colorectal cancer cells to confirm the results predicted in their RNAseq analysis (40). The analysis of 188 endogenous metabolites revealed that 97 of them were significantly changed between WT KRAS- or KRAS G13D-expressing cells, showing different metabolic profiles (40). The metabolic data revealed that KRAS G13D-expressing cells have an increased abundance of almost all amino acids, results that are consistent with the pathway analysis of up-regulated genes (40). In addition, this work showed a decrease in PC levels and an increase in carnitine and its esters in KRAS G13D-expressing cells (40). These findings are consistent with those previously published by Brunelli et al. concerning KRAS G12C (39), suggesting that these changes are not a codon-specific signature.

The results provided by omics profiling studies indicate that the differences in biological properties or downstream signaling pathways activation of distinct RAS proteins mutations are presumably consequences of their very specific transcriptomic, proteomic/phosphoproteomic and metabolomic profiles. The large amount of data provided by such profiles allows the comparison of different RAS mutant variants to determine their differences in a particular cancer or to provide important insights in the response to a specific treatment. Moreover, these studies identify hits that might be potential targets in therapy, as they are involved in numerous pathways previously described to be altered in cancer.

## RAS Mutated Variants at Non-canonical Codons

The most studied mutations in RAS genes are located at the canonical codons 12, 13, and 61. However, other mutations at non-canonical codons of RAS, such as 19, 22, 59, 117, or 146, have been described (6, 58–61). Both somatic as well as germline mutations at these codons have been reported. For example, NRAS A146T can be found in the leukemic cell lines NALM6 and ML-216, while HRAS K117N and A146T germline mutations have been identified in a small number of patients with Costello syndrome (62) and KRAS V14I in patients with Noonan syndrome (60). In addition, point mutations at codon 59 are commonly identified in the viral forms of HRAS and KRAS (58).

As non-canonical mutations have also been identified in patients' samples (59, 60, 62) and thus may be relevant for oncogenesis, functional and biochemical evaluation of these mutant protein have been performed in comparison with wild type RAS or other canonical RAS mutations (6, 59, 60).

### Transforming Potential

Feig and Cooper described two different HRAS non-canonical mutations, V14M and A146V, and assessed their transforming potential by their ability to form foci (58). Whereas, NIH3T3 fibroblasts expressing HRAS V14M had an indistinguishable foci formation ability compared to WT HRAS, HRAS A146V showed an increase in foci formation (58). This work also compared the transforming potential of WT HRAS and HRAS A59T and A59I, both of them identified as viral HRAS mutants defective in their autophoshporylation. HRAS A59T and A59I showed higher and lower transforming potential, respectively, compared to WT HRAS (58). The results concerning HRAS A59T are consistent with those previously published by Fasano et al. (63) and Lacal et al. (64), where HRAS A59T mutant protein was able to fully transform NIH3T3 mouse fibroblasts (64) and form foci compared to WT HRAS (Table 4) (63).

**Table 4:** Phenotypical and signaling differences among RAS proteins non-canonical mutations.

**Characteristic/mutation**	**HRAS** ** V14M**	**HRAS** ** A146T**	**HRAS** ** A59T**	**HRAS** ** A59I**	**KRAS** ** Q22K**	**NRAS** ** G60E**	**KRAS** ** T74P**	**KRAS** ** A146T**	**KRAS** ** L19F**	**KRAS** ** K117N**	**KRAS** ** R164Q**	**KRAS** ** A18D**
Transforming potential	No (58)	Yes (58)	High (58,63,64)	Low (58)	Low (65)	Yes (60)	Yes (60)	Yes (22,60)	High (59) Low (22)	Yes (22)	No (22)	
GTP binding	High (58)	High (58)	As WT (58)	As WT (58)	Yes (62)	High (60)	High (60)	High (60)	High (59) Yes (22)	Yes (22,62) High (6)	No (22)	As WT (6)
Instrinsic GTP hydrolysis		As WT (58)	Low (58)	Low (58)				Low (61)				
Nucleotide exchange rate		Fast (58)	Very fast (58,66)	As WT (58)				Very fast (61)				
Anchorage-independent growth					No (65)				Yes (59)	Yes (6)		No (6)
Migration										No (6)		No (6)
MEK activation						Low (60)	High (60)	Very high (60)				
ERK activation						High (60)	High (60)	High (60,61)		As WT (6)		As WT (6)
AKT activation										As WT (6)		As WT (6)
PDK activation										As WT (6)		As WT (6)

*Summary of RAS mutant proteins manifestations according to different studied characteristics and their ability to activate downstream pathways. Proteins activation was assessed by phosphorylation at different residues. WT, wild type; GTP, guanosine-5′-triphosphate; ERK, extracellular signal-regulated kinase; MEK, mitogen-activated protein kinase; AKT, protein kinase B; PDK, 3-phosphoinositide-dependent protein kinase*.

Later, the sequencing of different types of cancers revealed new mutations at the non-canonical codons 22, 60, 74, and 146 (60, 65). Tsukuda et al. analyzed the transforming potential and the proliferation rate of KRAS Q22K *in vitro* and *in vivo* (65). NIH3T3 mouse fibroblasts transfected with WT KRAS or KRAS Q22K were able to form few foci compared to the well-characterized activating mutation KRAS G12V. However, KRAS Q22K-expressing fibroblasts showed typical transformed cell morphology: small, spindle-shaped cells with no tight adherence (65). Moreover, cells expressing KRAS Q22K were able to grow under starving, while WT KRAS cells ceased to grow within 10 days under the same experimental conditions. However, neither WT or mutant KRAS showed tumor formation *in vivo* in 15 days (Table 4), whereas fibroblasts expressing KRAS G12V formed progressive tumors (65). These results indicate that KRAS Q22K is able to change mouse fibroblasts morphology but its transforming potential is not sufficient to develop tumors *in vivo*. In addition, Tyner et al. transfected A31 fibroblasts and murine bone marrow cells with WT or different KRAS and NRAS mutants (60). Whereas, WT KRAS- or NRAS-expressing cells exhibited few foci, indicating contact inhibited growth, NRAS G60E and KRAS T74P and A146T were able to form numerous foci (Table 4) (60).

Furthermore, Akagi et al. reported another non-canonical mutation at codon 19 of KRAS (59). To assess its transforming potential, three different characteristics were measured: cell morphology, proliferation and saturation density (59). The transfection of NIH3T3 fibroblasts with plasmids encoding WT KRAS or KRAS L19F showed that clones expressing this mutant protein were smaller and more rounded than those expressing WT KRAS. Moreover, whereas WT KRAS expressing cells ceased to grow under starved conditions, KRAS L19F clones were able to grow and had greater density that could be due to their small cell size and loss of contact inhibition (59). This work also studied the ability of the mutant KRAS L19F to form tumors *in vivo*, reporting that 75% of the KRAS L19F injected clones developed tumors in contrast to 13% of WT KRAS clones (Table 4) (59). Therefore, these results indicate that KRAS L19F has a higher proliferation capacity *in vitro* and *in vivo* compared to WT KRAS.

Smith et al. also assessed the transforming potential of the previously studied non-canonical mutations KRAS L19F and A146T and two new KRAS mutations, K117N and R164Q (22). The transduction of NIH3T3 fibroblasts with plasmids expressing these KRAS mutations and WT KRAS revealed that KRAS K117N and A146T enabled foci formation, whereas KRAS L19F only formed isolated foci (Table 4) (22). These findings contrast with those previously described by Akagi et al. (59) for codon 19, but in agreement with those published by Tyner et al. (60) for KRAS A146T.

These observations indicate that, similarly to mutations at codons 12, 13 and 61, mutations at non-canonical codons of the different RAS proteins display a diverse phenotype regarding their transforming potential.

### GTP Binding

Feig and Cooper determined the nucleotide binding affinities of WT HRAS and HRAS V14M and A146V (58). Whereas, WT HRAS showed affinity of for both GTP and GDP, the affinity of HRAS V14M and A146V for GTP and GDP were higher compared to WT HRAS (Figure 1A) (58). Moreover, this work also assessed the GDP-GTP exchange and the GTPase activity of HRAS A146V. This mutation showed a fast nucleotide exchange compared to WT HRAS but the same GTPase activity (Table 4), indicating that the transforming potential of HRAS A146V reported in this work was due to an increase in the speed of nucleotide exchange rather than any alteration in its GTPase activity (58). Moreover, the nucleotide binding affinity, nucleotide exchange rate and GTPase activity were also studied for HRAS A59T and A59I mutant proteins. Both HRAS A59T and A59I mutations and WT KRAS bound GTP and GDP. Regarding the nucleotide exchange, whereas HRAS A59I exhibited nearly the same exchange rate as WT HRAS, HRAS A59T mutation showed a rate 10-fold greater than WT HRAS. However, both HRAS A59T and A59I mutant proteins showed a reduction in their intrinsic GTPase activity (Table 4) (58). The results concerning HRAS A59T are consistent with the ones previously published by Lacal and Aaronson (66), who determined that HRAS A59T showed 3- to 9-fold greater nucleotide exchange than WT HRAS (66). All together, these results indicate that the transforming potential of HRAS A59T is due to a reduction in GTPase activity and an increase in nucleotide exchange. However, the inability of HRAS A59I to form foci reported in this work indicates that a reduction of the GTPase activity is not sufficient to confer transforming capacity (58), suggesting that changes in the nucleotide exchange rate are also important at this codon to acquire transforming capacities.

Akagi et al. studied the ability of RAS non-canonical mutations to bind GTP (59). KRAS L19F showed elevated RAS-GTP levels compared to WT KRAS, which was consistent with the *in vitro* and *in vivo* transforming potential of this KRAS mutant (59). Later on, experiments in HEK 293T/7 cells transfected with WT or mutants KRAS and NRAS revealed that NRAS G60E, KRAS T74P, and A146T had increased RAS-GTP levels compared to WT NRAS and KRAS (Figure 1A, Table 4) (3). The increase in KRAS T74P-GTP levels could be explained as the substitution of proline may disrupt the protein conformation involved in GTP hydrolysis, thus impairing GTP-GDP exchange (60). In addition, Janakiraman et al. showed that HEK 293FT cells expressing KRAS Q22K, E31K, K117N, and A146T were able to bind GTP (Table 4), with KRAS Q22K mutant variant showing the highest levels and KRAS E31K levels similar to WT KRAS, establishing the following hierarchy Q22K >> K117N ≈ A146T >> E31K (62). Later on, Smith et al. showed that KRAS L19F, K117N, and A146T were able to bind GTP, but WT KRAS and KRAS R164Q were not (Figure 1A, Table 4). These results are consistent with the transforming potential of these mutants reported in this work (22) and with the previously observed ability of KRAS L19F and A146T to bind GTP (59, 60, 62). In addition, Stolze et al. reported that KRAS A18D has a similar GTP-binding to WT KRAS and control cells, which only express endogenous KRAS, following EGF stimulation (6). In contrast, KRAS K117N mutant protein showed an increase in GTP-biding up to 5 to 6-fold compared to control cells (Figure 1A, Table 4) (6), which is consistent with the data reported by Janakiraman et al. (62).

Recently, in a 2019 study, Poulin et al. compared the nucleotide exchange and GTP hydrolysis between WT KRAS and KRAS A146T (61). The authors reported in this work that KRAS A146T had ~12-fold higher GDP dissociation rate than WT KRAS, a difference that was further increased by the addition of the GEF protein SOS1. The intrinsic GTP hydrolysis of KRAS A146T was reduced compared to WT KRAS (Table 4), while GAP-mediated GTP hydrolysis was only mildly impaired (61). These results are consistent with those published previously (22, 60, 62). Therefore, the ability of KRAS A146T to form foci reported by Tyner et al. and Smith et al. (22, 60) might be due to an increase in the intrinsic and GEF-mediated nucleotide exchange rather than a loss of GAP-mediated exchange (61).

### Anchorage-Independent Growth and Migration

Using colony formation as a measurement of anchorage-independent growth, Tsukuda et al. have shown that KRAS Q22K formed only few colonies in soft agar, similar to WT KRAS (Table 4) (65), indicating that this mutation cannot grow independent of anchorage, results that are in agreement with its inability to form tumors *in vivo*. Later, Akagi et al. reported that 9.2% of NHI3T3 cells expressing KRAS L19F were able to form colonies, while fibroblasts expressing WT KRAS failed to do so (Table 4) (59), consistently with the transforming potential assessed in this study. In addition, Stolze et al. reported that MCF10A breast cancer cells ectopically expressing KRAS A18D at physiological levels were unable to form colonies in soft agar (6). However, KRAS K117N expressing cells displayed a slight increase in colony formation compared to control cells expressing endogenous KRAS (6). In addition, KRAS A18D- and K117N-expressing cells showed no increase in their migration abilities compared to WT KRAS or control cells when they are expressed at physiological levels (Table 4) (6).

### Downstream Pathways Activation and Outcome

Several studies assessed the activation of RAS downstream pathways by non-canonical mutations (6, 60, 61). For example, Tyner et al. studied the activation of the MAPK pathway by MEK and ERK phosphorylation status (60). Compared to WT NRAS, HEK 293T/17 cells expressing NRAS G60E showed an increase in ERK but not in MEK phosphorylation. In the case of KRAS T74P and A146T, both mutant proteins increased ERK phosphorylation levels in comparison to WT KRAS, but KRAS A146T showed higher MEK activation than KRAS T74P and WT KRAS (Figure 1B, Table 4) (60). In addition, Stolze et al. reported that MCF10A cells expressing KRAS A18D or K117N at physiological levels did not show higher phosphorylation levels of ERK, PDK, and AKT compared to WT KRAS or control cells expressing endogenous KRAS (Figure 1B, Table 4) (6). However, either the physiological expression or overexpression of KRAS K117N increased the activation of RPS6 compared to WT KRAS and control cells after the addition of EGF (Table 4) and thus, this mutant enabled the activation of the mTOR pathway (6). In addition, Poulin et al. analyzed the activation of the RAS downstream pathways assessing the phosphoproteome of WT KRAS and KRAS A146T (61). KRAS A146T mutant protein expressed in the colon increased the phosphorylation level of ERK1/2 compared to WT KRAS, but less than KRAS G12D (Table 4) (61). Therefore, KRAS A146T seems to activate the MAPK pathway less strongly compared to KRAS G12D. However, the inhibition of this signaling pathway reduced the proliferation in the colonic epithelium, indicating that the activation of the MAPK pathway at low levels is sufficient to increase the proliferation rate in this tissue (61).

In addition to *in vitro* studies which analyzed RAS mutations at non-canonical codons, *in vivo* xenograft models have also been employed to study the activation of downstream signaling pathways and related outcomes (61, 62). For example, Janakiraman et al. showed >95% decrease in ERK phosphorylation 6h after the inhibition of the MAPK pathway. Moreover, this inhibition was also associated with a downregulation of cyclin D1, an increase in p27 expression and hypophosphorylation of Rb (62).

Poulin et al. studied the phenotype that ensues from the expression of KRAS A146T in the colonic epithelium, hematopoietic stem cells and pancreas of genetically engineered mice (61). In the colonic epithelium, KRAS A146T caused a moderate hyperplasia and an intermediate proliferation between KRAS G12D and WT. The expression of KRAS A146T in hematopoietic stem cells led to a myelodysplastic syndrome/myeloproliferative neoplasm with a delayed onset compared to mice expressing KRAS G12D in the same cells, and these animals died with severe anemia and splenomegaly at an older age than KRAS G12D-expressing mice (61). However, when KRAS A146T was expressed in the pancreas, mice showed no evidence of pancreatic intraepithelial neoplasia at 2 months of age. Even the induction of acute pancreatitis was not sufficient to induce pancreatic neoplasia, suggesting that this mutation does not alter pancreatic homeostasis (61).

### Transcriptomics and Proteomics

Differences in overall mRNA and protein expression among RAS non-canonical codons have been described (22, 61). Smith et al. performed a hierarchical clustering of transcriptomic data of WT KRAS, KRAS canonical mutations G12V, G12C, G12D, and G13D and KRAS non-canonical mutations L19F, K117N, A146T, and R164Q (22). The analysis revealed two different clusters: WT KRAS and the codon 12 mutations clustered in one group (“cluster one”) while the codon 13 and non-canonical mutations clustered in a second group (“cluster two”), indicating that non-canonical mutations displayed similar gene expression profile to KRAS G13D. Despite previous results of this study showing that KRAS R164Q had a similar transforming potential to WT KRAS (22), this mutation was grouped in the “cluster two,” suggesting an attenuated transforming potential. In addition, KRAS L19F and R164Q formed a transcriptomic subcluster within the “cluster two,” suggesting that these two mutations are different from KRAS G13D, K117N, and A146T (22). Furthermore, Smith et al. analyzed the expression of genes involved in signal transduction, cytoskeleton remodeling and cell adhesion (22). Despite the few changes in gene expression induced by KRAS R164Q, there were examples of genes whose expression was induced by all mutants, such as the protein tyrosine phosphatase *PTPRE* and the RHO GTPase-activating protein *ARHGAG6* (22). Moreover, genes induced or repressed by all of the mutants except KRAS R164Q were identified, including the MAPK phosphatases *DUSP4* and *DUSP6*, the RHO guanine-exchange factor *NGEF*, the cell adhesion molecule *CEACAM1* and the plasminogen activator inhibitor *SERPINB2*. Interestingly, “cluster one” but not “cluster two” KRAS mutants differentially expressed some genes, for example *VEGFA, PAK3*, or *PIM1*; and “cluster two” but not “cluster one” mutants showed a different expression of *IGF1R* and *CREB1* among other genes. Additional genes, such as *E2F2, SLC2A1*, or *JUN*, were differentially regulated by all the analyzed mutants (22).

Poulin et al. studied the proteome and phosphoproteome of colon, pancreas and spleen from mice expressing WT KRAS or the mutant proteins KRAS G12D and A146T (61). The data derived from each tissue revealed that the two mutant variants and WT KRAS clustered separately. The collective analysis of all data showed that samples from the same tissue cluster together regardless of the KRAS mutation and samples expressing KRAS A146T tended to cluster closer to the ones expressing WT KRAS (61), suggesting that WT KRAS and KRAS A146T display similar proteomic and phosphoproteomic profiles in these tissues. Moreover, the same authors uncovered the enriched biological pathways in KRAS G12D or A146T using the dataset of each tissue analyzed (61). In the colon dataset, KRAS G12D and A146T differentially regulated the majority of the enriched pathways, such as the calcium signaling pathway. Similar to the colon-associated data, the majority of pathways enriched in the pancreas dataset were discordantly regulated by both mutations. Interestingly, whereas the nitrogen metabolism pathway was up-regulated by KRAS G12D and A146T in colon, the same pathway was down-regulated in the pancreas. In the spleen dataset, KRAS G12D and A146T showed no pathways differentially regulated by the two mutants compared to WT KRAS (61). Conclusively, KRAS G12D and A146T differentially regulate downstream signaling pathways, depending also on the tissue where these mutant proteins are expressed.

All together, these results suggest that, similarly to mutations at codons 12, 13, and 61, mutations at non-canonical codons of RAS proteins display different biological manifestations that relate to their transforming potential and GTP binding. Moreover, these mutations activate differently RAS downstream signaling pathways and alter genes and proteins expression compared to the WT protein. However, non-canonical mutations are less studied compared to mutations at codons 12, 13, and 61, despite the fact that they have been described in patients' samples. Therefore, it is of an immense interest to continue studying their biological characteristics *in vitro* and *in vivo* to uncover more over the properties of those uncommon variants and their relevance to RAS-related oncogenesis.

## RAS Proteins Mutations Affect Treatment Responses

KRAS is the most frequently mutated RAS protein in cancer (5) and therefore the most studied in clinical trials for different therapy regimen ^1^. The association between treatment responses and survival in patients carrying KRAS mutant variants has been studied since the late 1990s (67). For example, Keohavong et al. reported that lung cancer patients carrying the KRAS G12V or G12R mutations had a shorter overall survival (OS) compared to those with WT KRAS tumors, while KRAS G12D-carrying patients showed longer survival (67). Regarding treatment response, Petrelli et al. reported in a meta-analysis of 12 colorectal cancer clinical trials that patients carrying WT KRAS had a better response rate (RR) to chemotherapy plus bevacizumab than those harboring KRAS-mutated tumors (68). Of particular importance, Allegra et al. reported in their retrospective study that colorectal cancer (CRC) patients carrying KRAS mutant variants do not benefit from the anti-EGFR antibodies cetuximab and panitumumab (69). However, it has also been demonstrated that about 10% of the patients with KRAS-mutated tumors can respond to anti-EGFR therapy and about 15% have long-term disease stabilization (70). This subchapter discusses in detail the relation between KRAS mutant variants and survival and treatment response of patients with various cancer types that are particularly prone to carry KRAS mutations.

### Colorectal Cancer

Approximately 40% of CRC cases harbor KRAS mutations at codons 12, 13, and 61, resulting mainly in the KRAS G12D, G12V, and G13D variants (2, 5). Almost already 10 years ago, De Rock et al. analyzed whether the presence of the KRAS G13D mutant variant is associated with treatment response or survival of CRC patients (70). As KRAS G13D has been reported to exhibit weaker transforming potential than KRAS codon 12 mutant variants (6, 22, 25), De Rock et al. hypothesized that patients harboring KRAS G13D mutation might have a better outcome after cetuximab treatment compared to patients carrying other KRAS mutant variants. To confirm this hypothesis, 579 patients with varying KRAS status who had chemotherapy-refractory metastatic colorectal cancer (mCRC) were divided into two different treatment lines: cetuximab only and cetuximab plus chemotherapy (70). Compared to other KRAS mutant variants, patients carrying KRAS G13D mutant variant who received cetuximab treatment, either alone or in combination with chemotherapy, had longer OS (median 7.6 months vs. median, 5.7 months, HR, 0.50) and progression-free survival (PFS) (median 4.0 months vs. median, 1.9 months, HR, 0.51). However, no significant differences in OS or PFS were identified in KRAS G13D patients compared to those carrying the WT KRAS (70). Similarly, patients with KRAS G13D-expressing tumors receiving the combination treatment of cetuximab plus chemotherapy showed longer OS and PFS than patients carrying other KRAS mutant variants (OS: median, 10.6 months vs. 7.4 months, HR, 0.46; PFS: median, 4.1 months vs. 2.8 months, HR, 0.49). No differences between KRAS G13D and WT KRAS regarding OS and PFS were identified, results also reported in the “cetuximab only” group (70). These results confirmed that patients carrying KRAS G13D benefited from cetuximab treatment compared to those carrying other KRAS mutant variants, which may be explained by the weak transforming potential showed *in vitro* (70). This work also compared the RRs of KRAS G13D patients in the different treatment groups (70). Patients carrying KRAS G13D mutant variant receiving the combination of cetuximab plus chemotherapy showed higher but not statistically significant RR compared to patients with other KRAS mutations. However, patients carrying WT KRAS in the cetuximab plus chemotherapy treatment arm showed higher RR than those with tumors expressing KRAS G13D, but this difference is not statistically significant when WT KRAS patients are compared to KRAS G13D patients receiving cetuximab only (70). In addition, De Rock et al. studied the *in vivo* response to cetuximab (70). Cetuximab inhibited the growth of tumors harboring WT KRAS or KRAS G13D, showing a similar response to the treatment; however, the treatment did not affect the growth of KRAS G12V-expressing tumors (70), suggesting that KRAS codon 12 mutant variants are resistant to cetuximab.

Later, Tepjar et al. combined the data of 1,378 mCRC patients included in the previous clinical trials (71). In this work, patients carrying the KRAS G13D mutant variant had additional benefit from chemotherapy plus cetuximab than from chemotherapy alone. These patients showed higher PFS (median 7.4 vs. 6.0 months; HR, 0.47) and tumor response (median 40.5 vs. 22.0% months; OR, 3.38), but not OS (median 15.4 vs. 14.7 months; HR, 0.89), when cetuximab was added to the chemotherapy regimen. These results could partially be explained by the worse prognosis of KRAS G13D patients in the control arm (71). Opposite to KRAS G13D, patients carrying the KRAS G12V mutant variant receiving chemotherapy plus cetuximab showed worse PFS than those receiving chemotherapy only (71). When only the chemotherapy plus cetuximab treatment arm was considered, patients carrying G13D or G12V mutant variants showed a similar OS, which was markedly lower than those patients with WT KRAS tumors (71). Within the chemotherapy only arm, patients with KRAS G13D mutant tumors tended to have worse, but not statistically significant, PFS and OS compared to those harboring tumors with other KRAS mutant variants (PFS: HR, 1.49; OS: HR, 1.25). However, patients with KRAS G12V tumors did not showed worse PFS or OS compared to other KRAS mutant tumors (PFS: HR, 0.77; OS: HR, 1) (71).

Similarly, Fiala et al. studied KRAS mutations at codons 12 and 13 of mCRC patients treated with the anti-angiogenic antibody bevacizumab who previously have received different chemotherapeutic regimen (72). Patients carrying mutant variants at codons 12 or 13 had shorter OS and PFS than those with WT KRAS (72). When each KRAS mutant variant was analyzed independently, patients carrying KRAS G12V or G12A had shorter PFS and OS compared to WT KRAS patients (PFS: HR = 2.18; OS: HR = 2.58) (72).

The impact of KRAS mutant variants have been analyzed not only in relation to chemotherapy, but also regarding surgery as a treatment strategy (73, 74). Mangonis et al. studied the outcome of CRC patients carrying KRAS mutations after curative intent liver resection due to liver metastasis (73). Patients carrying KRAS mutant variants at codon 12 (G12V, G12D, G12C, G12S, and G12A) or KRAS G13D had no significant differences in 5-year recurrence-free survival (RFS) compared to WT KRAS patients (*p* = 0.57). Moreover, none of the aforementioned mutant variants were associated with worse RFS than WT KRAS (73). In addition, patients carrying any KRAS codon 12 mutant variant had worse 5-year OS compared to WT KRAS patients (HR, 1.7), while KRAS G13D patients had no differences in OS compared to those carrying WT KRAS (HR, 1.47) (73). When each KRAS mutant variant was analyzed independently, KRAS G12V and G12S were associated with 2- to 3-fold increase risk for long-term death compared to WT KRAS patients. In addition, patients carrying KRAS G12V, G12S, and G12C had a higher risk of death after recurrence compared to those harboring WT KRAS who recurred (73). Recently, Hayama et al. analyzed 200 CRC patients who underwent curative resection (74). Analysis of relapse-free survival revealed that a small proportion of patients carrying KRAS mutant variants G12D, G12V, G12C, G12A, G12S, or G13D reached the 3-year relapse-free survival endpoint compared to those carrying WT KRAS (69.7 vs. 82.1%, respectively; *p* = 0.01). Moreover, patients carrying KRAS G12V or G12C had a higher risk of long-term recurrence than those with WT KRAS tumors or KRAS G12A, G12D, or G12S-mutated CRC (74).

Independently of the type of treatment (chemotherapy or surgery), CRC patients carrying KRAS G13D mutant variant seem to show no significant differences in PFS, OS or RFS as compared to WT KRAS across these various studies. However, patients harboring KRAS codon 12 mutant tumors generally had worse PFS, OS, RFS and RR compared to patients with WT KRAS tumors. This could be potentially explained by the fact that KRAS codon 12 mutant variants, especially the G12V and G12D mutations, have been reported to have a very high transforming potential and a low GTP intrinsic and GAP-mediated GTP hydrolysis (6, 22, 25). Importantly, this also correlates with the *in vivo* animal models findings reported by De Rock et al. regarding KRAS G12V and its resistance to cetuximab (70) and with preclinical results by Leiser et al. on KRAS G12V, G12D, and G13D mutant variants conferring resistance to two different MET inhibitors (75).

### Non-small Cell Lung Cancer (NSCLC)

KRAS mutations, mainly KRAS G12C, G12D, and G12V, are observed in 20–30% of NSCLC patients, predominantly in patients with adenocarcinomas (2, 76). Subsequently, the association between KRAS mutant variants and treatment survival in NSCLC has also been extensively studied (23, 76, 77). For example, Ihle et al. reported that patients with refractory NSCLC carrying KRAS G12C or G12V mutant variants showed a statistically significant decrease in PFS (median survival = 1.84 months; *p* = 0.046) compared to other KRAS mutant variants (G12A and G12D) (median survival = 3.35 months) or WT KRAS (median survival = 1.95 months) (23). This association was more pronounced in patients receiving sorafenib whereas no statistically significant association was identified between patients harboring KRAS G12C or G12V mutations and PFS in either erlotinib or bexarotene plus erlotinib treatment groups (23). Later on, Mellema et al. analyzed whether there was an association between KRAS codon 12 mutant variants and OS, PFS and RR in 464 advanced NSCLC patients who received platinum-based chemotherapy as a first-line treatment (76) Patients in this study were treated with different agents (pemetrexed, gemcitabine, taxane or bevacizumab) in addition to the previously administrated platinum treatment (76). Interestingly, patients carrying KRAS G12V mutant variant showed a higher RR when treated with taxanes than those harboring the same mutant variant but treated with pemetrexed or gemcitabine. However, KRAS G12V patients in the taxanes group had longer, but not statistically significant, PFS and OS compared to patients carrying the same mutant variant but treated with pemetrexed or gemcitabine. Moreover, patients carrying KRAS G12C or G12D mutant variants had similar RR, PFS and OS within all treatment groups (76). In addition, this work assessed the PFS and OS among KRAS G12C, G12V, and G12D mutant variants independently of the received treatment, showing no differences (PFS: median 4.9, 4.8, and 4.3 months for G12C, G12V, and G12D, respectively (*p* = 0.45); OS: median 10.4, 8.0, and 8.3 months for G12C, G12V, and G12D, respectively (*p* = 0.46) (76).

Interestingly, Renaud et al. also analyzed KRAS-mutated patients with NSCLC who received platinum-based chemotherapy as first-line treatment, with similar treatment arms: pemetrexed, vinorelbine, gemcitabine, taxane, or bevacizumab (77). Amino acid substitutions in KRAS did not affect patients' OS even when differences in treatment were considered (77). When assessing the time to progression (TTP), treatments with vinorelbine and taxane were associated with a better TTP (HR, 0.76 and 0.32, respectively) compared to gemcitabine and pemetrexed treatment arms. However, KRAS mutational status was not a significant predictor of TTP, despite the fact that patients carrying KRAS G12D or G12V mutant variants tended to have a better, but not statistically significant, TTP compared to those carrying KRAS G12C. This tendency was also observed in patients carrying KRAS G13D mutant variant when treated with taxane. Interestingly, all KRAS mutant variants identified in this study were associated with worse TTP when treated with bevacizumab compared to other treatment regimen (77).

To summarize, in the case of NSCLC, contradictory results addressing the impact of KRAS different mutations in clinical settings are being reported. While Ihle et al. and Mellema et al. reported that patients carrying KRAS G12V mutant variant showed longer OS and PFS when treated with taxanes compared to other treatment regimen (23, 76), Renaud et al. reported no differences among the codon 12 amino acid substitution considering the various treatment arms, including taxanes (77). Moreover, this work showed that patients with KRAS G12D or G12V-expressing tumors tended to have better TTP than those with KRAS G12C tumors (77), which contrasts with the higher PFS and OS reported by Mellema et al. for KRAS G12C patients (76). Additional studies would thus be needed to allow for consistent conclusions and potential clinical implementation of such findings.

### Pancreatic Ductal Adenocarcinoma

Pancreatic adenocarcinoma is considered one of the most aggressive forms of cancer. KRAS mutations are carried approximately by 90% of the patients and can be detected at both early and chronic stage of the disease (5). Among all the possible KRAS mutant variants, the most frequently observed in the pancreatic ductal adenocarcinoma (PDAC) are KRAS G12D and G12V (2).

As previous studies in colorectal and lung cancer have demonstrated that KRAS status influences patients prognosis (23, 71), Bournet et al. studied whether KRAS codon mutant variants were associated with the OS in 219 patients with metastatic or locally advanced PDAC (78). This work reported no differences in OS between patients carrying WT KRAS or codon 12 mutations (KRAS G12D, G12V, and G12R). However, KRAS G12D patients showed a decreased OS compared to KRAS G12V or G12R patients (78). These results are in agreement with the previously published data by Boeck et al. who reported median OS of 5.3 months for patients carrying KRAS G12D, 6.6 months for those with the KRAS G12V mutation and 7.7 months for patients with tumors harboring the G12R KRAS mutation (79). Moreover, Bournet et al. showed that among all the 162 patients who received chemotherapy, those carrying the KRAS G12D mutant variant had a worse prognosis compared to those with KRAS G12V or G12R. However, patients carrying KRAS codon 12 mutant variant in the chemotherapy treatment subgroup showed no difference in OS compared to WT KRAS patients. Similar results were also reported in a subgroup of 119 patients who received gemcitabine as first-line treatment (78).

Collectively, data summarized in this chapter suggest that CRC patients carrying the KRAS G13D mutant variant show a similar treatment response as those carrying WT KRAS and, thus, will benefit from anti-EGFR therapy. Interestingly, KRAS codon 12 mutants showed different outcome results depending on the cancer type and treatment employed. These results are reflected in KRAS G12V patients, who showed worse PFS when treated with cetuximab but an increase in this endpoint with taxanes treatment. However, of course, such generalization of the results should be taken into consideration only very carefully due to the possible differences in basic patients' characteristics, the sample size and the treatment regimen in each study.

## Final Remarks

In these emerging times of personalized medicine, it is highly anticipated that detailed knowledge of cancer genomic landscapes may improve treatments, resulting ultimately in a significant increase of patients' survival. The members of the RAS subfamily of GTPases, which includes KRAS, HRAS, and NRAS, are frequently mutated in cancer. KRAS is often altered in pancreatic carcinoma, colorectal tumors and lung malignancies and *HRAS* mutations are common in dermatological malignancies and head and neck cancers whereas *NRAS* alterations in melanomas and in hematopoietic malignancies. Despite the differences in mutations rates at each codon, the three RAS proteins are usually mutated at the canonical codons 12, 13, and 61. However, other mutant variants have been described at non-canonical codons such as 19, 59, 117, and 146, illustrating the complexity that is affiliated with thes oncogenes. As canonical codons are located in the homologous amino-acid region, shared by all RAS proteins, it could be postulated that their effect on the protein function is equivalent. However, various experimental lines of evidence summarized in this review have demonstrated that not all RAS mutant variants display the same biological and biochemical properties, suggesting that tumors harboring different RAS mutations may behave differently according to the expressed RAS mutant variant. Therefore, detailed knowledge about the biological and biochemical properties of each RAS mutant variant *in vitro* and *in vivo* seems to be essential to help understand the biology of the particular treatment and possibly predict patients' treatment response and survival.

At the same time, the vast majority of preclinical as well as clinical studies are mostly focused on RAS canonical mutations. However, rare RAS mutant variants seem to display similar differences in their biological properties and downstream signaling activation and, thus, their more extensive studying could help to better understand the behavior of RAS-expressing tumors. Screening and additional biologic characterization of these non-canonical RAS mutations should also be considered in clinical practice as mutational analyses of codons 12, 13, and 61 only may misclassify patients that could benefit from particular anti-cancer therapies.

## Author Contributions

CM-M, YZ, and MM designed the study, edited, and approved the final version. CM-M searched the literature and drafted the manuscript. YZ and MM supervised the study.

### Conflict of Interest

The authors declare that the research was conducted in the absence of any commercial or financial relationships that could be construed as a potential conflict of interest.

## Footnotes

^1^https://clinicaltrials.gov
